# Prescrição de Exercícios Aeróbicos: O Teste de Fala Supera o Teste de Caminhada de 6 Minutos

**DOI:** 10.36660/abc.20230636

**Published:** 2023-10-16

**Authors:** Márcio Garcia Menezes, Vitor Augusto Fronza, Leandro Franzoni

**Affiliations:** 1 Universidade Federal do Rio Grande do Sul Porto Alegre RS Brasil Universidade Federal do Rio Grande do Sul, Porto Alegre, RS – Brasil; 2 Universidade Federal do Rio Grande do Sul Porto Alegre RS Brasil Universidade Federal do Rio Grande do Sul – Grupo de Pesquisa em Cardiologia do Exercício (CardioEx), Porto Alegre, RS – Brasil

**Keywords:** Aptidão Cardiorespiratória, Resistência Física, Teste de Caminhada, Reabilitação Cardiovascular, Qualidade de Vida

As doenças cardiovasculares (DCV) representam uma realidade preocupante, levando a uma elevada taxa de morbidade e mortalidade em muitos países, com um total global de 18 milhões de mortes. Além disso, estas condições representam encargos econômicos e sociais substanciais.^1-3^

A reabilitação cardiovascular é uma ferramenta promissora em beneficiar pacientes com DCV, por meio de exercícios aeróbicos (EA) como aliados essenciais na melhoria da aptidão cardiorrespiratória, resistência muscular, função cardiovascular e qualidade de vida geral.^1-3^ Segundo a Sociedade Brasileira de Cardiologia, a recomendação geral para a prática de exercícios físicos é de 150 minutos semanais, distribuídos entre três a cinco sessões.^2^ Um dos principais objetivos da reabilitação cardiovascular é melhorar a aptidão cardiorrespiratória através da incorporação de EA.^1,3^ Além disso, a prática criteriosa de atividade física visa mitigar a hipertensão, o risco de eventos cardiovasculares e a mortalidade geral em pacientes cardiovasculares.^1-3^

Vale ressaltar que métodos individualizados para prescrição de EA, como por exemplo, através do consumo máximo de oxigênio e limiares ventilatórios identificados por meio do teste cardiopulmonar de exercício (TCPE), são amplamente reconhecidos. Contudo, nos países em desenvolvimento, onde os custos associados a estes testes podem ser proibitivos e existe escassez de profissionais altamente qualificados, a prescrição de EA é muitas vezes baseada em testes de campo, como o Teste de Caminhada de 6 Minutos (TC6) e o Teste de Fala (TF).

Neste cenário, foi o que Althoff et al. 2023 ^3^ fizeram em seu recente artigo publicado nos Arquivos Brasileiros de Cardiologia. Este estudo buscou analisar a frequência cardíaca (FC) durante as etapas do TF e no pico do TC6 como parâmetro para prescrição de EA, comparada com a FC no primeiro e segundo limiares ventilatórios (LV1 e LV2) do TCPE. Para a realização do estudo foram avaliados 22 pacientes com idade entre 40 e 80 anos com diagnóstico de DCV crônica clinicamente estável. Participaram de três dias de avaliação: o primeiro dia consistiu em anamnese (entrevista estruturada e medidas antropométricas, incluindo índice de massa corporal e avaliação clínica) e TCPE, considerado padrão ouro para avaliação da função cardiopulmonar e prescrição de intensidade de exercício. Isso permitiu identificar as variáveis mais adequadas e recomendadas, como consumo de oxigênio de pico (VO_2_pico), carga horária, FC máxima, reserva de FC e VT1 e VT2.^1,3-5^O segundo dia envolveu o TC6 e o terceiro dia incluiu o TF, que é validado e acessível, baseado em um protocolo de carga incremental e utiliza a percepção de conforto de fala como marcador de intensidade do exercício.^3^

Em relação aos resultados, vale ressaltar que a FC no LV1 foi semelhante à FC no TF (p = 0,987) e TF± (p = 0,154) e correlacionou-se moderadamente com TF+ (r = 0,479, p = 0,024). A FC no VT2 foi semelhante à do TF (p = 0,383), mas apresentou forte correlação (r = 0,757, p < 0,001). A FC máxima durante o TC6 foi significativamente diferente da FC em TF+, TF± e VT1 (p = 0,001, p = 0,005 e P < 0,001, respectivamente), mas semelhante ao TF (p = 0,68).

Por meio desses resultados, observamos a possibilidade de identificar correlações no TF entre a FC no LV1 e TF+ e TF±, indicando semelhanças entre a FC e o VO_2_pico nos estágios TF e VT em pacientes com DCV. Esses achados sugerem que os estágios do TF podem ser empregados na prescrição de EA, alinhando-se com algumas evidências existentes na literatura científica.^3,6,7-9^ Além disso, o estudo demonstrou que o TC6 não é tão adequado para prescrição de EA quanto o TF. Apesar do conhecimento de que o TC6 é um teste submáximo, seu uso pode ser incentivado em um ambiente de baixo custo, uma vez que ainda fornece informações clinicamente relevantes, mesmo que não seja projetado especificamente para prescrição de exercícios.O aspecto distintivo deste estudo é o fornecimento de uma alternativa custo-efetiva que pode auxiliar na prescrição de exercícios no contexto da reabilitação cardíaca.

Este estudo sugere que o TF pode ser uma alternativa viável para a prescrição de EA, particularmente em ambientes com recursos limitados. Demonstrou correlações entre os estágios do TF e os limiares derivados do TCPE, indicando sua utilidade potencial na prescrição de EA para pacientes com DCV. Além disso, o TC6, embora menos adequado, continua a ser uma opção custo-efetiva, fornecendo informações clínicas valiosas em contextos de reabilitação cardíaca. Este estudo oferece informações valiosas para a prescrição de EA em ambientes de saúde desafiadores.
