# Supplementary material for: Prescrição de Exercícios Aeróbicos: O Teste de Fala Supera o Teste de Caminhada de 6 Minutos
Source: Arq Bras Cardiol. 2023 Oct 16;120(9):e20230636. [Article in Portuguese] doi: 10.36660/abc.20230636 (PMC10586818; doi:10.36660/abc.20230636)
Supplement: Translation

# Translation: Aerobic Exercise Prescription: The Talk Test Rises Over the 6-Minute Walk Test

**Authors:** Márcio Garcia Menezes, Vitor Augusto Fronza, Leandro Franzoni

**Affiliations:** 1 Brazil Universidade Federal do Rio Grande do Sul, Porto Alegre, RS – Brazil; 2 Brazil Universidade Federal do Rio Grande do Sul – Grupo de Pesquisa em Cardiologia do Exercício (CardioEx), Porto Alegre, RS – Brazil

**Keywords:** Cardiorespiratory Fitness, Physical Endurance, Walk Test, Cardiovascular Rehabilitation, Quality of Life

Cardiovascular diseases (CVDs) represent a concerning reality, leading to a high morbidity and mortality rate in many countries, with a global total of 18 million deaths. Moreover, these conditions pose substantial economic and social burdens.^1-3^

Cardiovascular rehabilitation holds significant promise in benefiting patients with CVDs, with aerobic exercises (AE) as pivotal allies in improving cardiorespiratory fitness, muscular endurance, cardiovascular function, and overall quality of life.^1-3^ According to the *Sociedade Brasileira de Cardiologia*, the general recommendation for engaging in physical exercise is 150 minutes per week, distributed across three to five sessions.^2^ One of the foremost objectives in cardiovascular rehabilitation is to enhance cardiorespiratory fitness through the incorporation of AE.^1,3^ Furthermore, the judicious practice of physical activity aims to mitigate hypertension, the risk of cardiovascular events, and overall mortality in cardiovascular patients.^1-3^

It is worth noting that individualized methods for prescribing AE, such as maximal oxygen consumption and ventilatory thresholds identified through cardiopulmonary exercise testing (CPET), are widely recognized. However, in developing countries, where the costs associated with these tests can be prohibitive, and a shortage of highly skilled professionals exists, the prescription of aerobic exercises is often based on field tests, such as the 6-Minute Walk Test (6MWT) and the Talk Test (TT).

In this scenario, Althoff et al. 2023 ^3^ published their recent article in *Arquivos Brasileiros de Cardiologia*. This study sought to analyze heart rate (HR) during the stages of the TT and at the peak of the 6MWT as a parameter for prescribing AE, compared with HR at the first and second ventilatory thresholds (VT1 and VT2) of the CPET. To conduct the study, 22 patients aged between 40 and 80 years with a diagnosis of clinically stable chronic CVD were evaluated. They attended three assessment days: the first day consisted of anamnesis (structured interview and anthropometric measurements, including body mass index and clinical evaluation) and CPET, considered the gold standard for assessing cardiopulmonary function and prescribing exercise intensity. This allowed for the identification of the most appropriate and recommended variables, such as peak oxygen uptake (VO_2_peak), workload, maximum HR, HR reserve, and VT1 and VT2.^1,3-5^ The second day involved the 6MWT, and the third day included the TT, which is validated and accessible, based on an incremental load protocol and uses speech comfort perception as an exercise intensity marker. ^3^

Regarding the results, it is worth noting that the HR at VT1 was similar to the HR at TT (p = 0.987) and TT± (p = 0.154) and moderately correlated with TT+ (r = 0.479, p = 0.024). The HR at VT2 was similar to that at TT (p = 0.383) but showed a strong correlation (r = 0.757, p < 0.001). The peak HR during the 6MWT was significantly different from the HR at TT+, TT±, and VT1 (p = 0.001, p = 0.005, and p < 0.001, respectively) but similar to TT (p = 0.68).

Through these results, we observe the possibility of identifying correlations in the TT between HR at VT1 and TT+ and TT±, indicating similarities between HR and VO_2_peak in TT and VT stages in patients with CVDs. These findings suggest that TT stages can be employed for AE prescription, aligning with some existing evidence in the scientific literature.^3,6-9^Furthermore, the study demonstrated that the 6MWT is not as suitable for AE prescription as the TT. Despite the knowledge that 6MWT is a submaximal test, its use can be encouraged in a low-cost setting, given that it still provides clinically relevant information, even if it is not specifically designed for exercise prescription. The distinctive aspect of this study is the provision of a cost-effective alternative that can assist in exercise prescription within the context of cardiac rehabilitation.

This study suggests that TT may be a viable alternative for AE prescription, particularly in resource-limited settings. It demonstrated correlations between TT stages and CPET-derived thresholds, indicating its potential utility in AE prescription for CVD patients. Additionally, the 6MWT, while less suitable, remains a cost-effective option, providing valuable clinical information in cardiac rehabilitation contexts. This study offers valuable insights for AE prescription in challenging healthcare environments.
